# Implementation of MenACWY vaccination because of ongoing increase in serogroup W invasive meningococcal disease, the Netherlands, 2018

**DOI:** 10.2807/1560-7917.ES.2018.23.16.18-00158

**Published:** 2018-04-19

**Authors:** Mirjam J Knol, Wilhelmina LM Ruijs, Laura Antonise-Kamp, Hester E de Melker, Arie van der Ende

**Affiliations:** 1 Center for Infectious Disease Control, National Institute for Public Health and the Environment, Bilthoven, the Netherlands; 2 Netherlands Reference Laboratory for Bacterial Meningitis, Academic Medical Center, Amsterdam, the Netherlands

**Keywords:** meningococcal disease, vaccine-preventable diseases, vaccines and immunisation, public health policy, Neisseria meninigitidis, bacterial infections

## Abstract

The annual incidence rate of serogroup W invasive meningococcal disease in the Netherlands increased from < 0.05/100,000 (n < 10) before 2015 to 0.5/100,000 (n = 80) in 2017. Most isolates (94%) belong to clonal complex 11. The incidence rate is highest among  < 5 year-olds and 15–24 year-olds. The case fatality rate was 12% (17/138) in 2015–2017. From May 2018, MenACWY vaccination replaces MenC vaccination at age 14 months and from October 2018, 13–14 year-olds are offered MenACWY vaccination.

The Netherlands currently experiences an ongoing increase of serogroup W invasive meningococcal disease (IMD). Our aim is to describe the epidemiological and microbiological characteristics of the current increase and the implementation of MenACWY vaccination in the Netherlands in 2018.

## Ongoing increase of serogroup W invasive meningococcal disease

**Figure 1:**
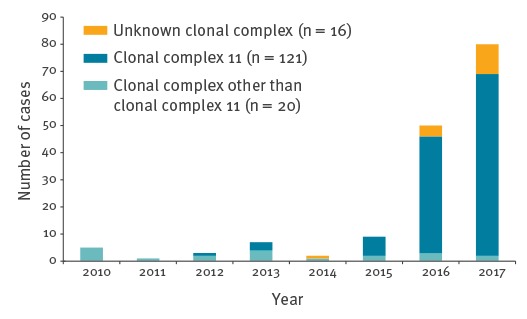
Number of cases of serogroup W invasive meningococcal disease by clonal complex in the Netherlands, 2010–2017 (n = 157)

In the Netherlands, serogroup W IMD was very rare before 2015 with an average annual incidence rate of 0.02 cases per 100,000 and on average four cases per year from 2010 up to 2014 (Figure 1). From 2015 the incidence rate started to increase reaching 0.5 per 100,000 in 2017 (n = 80). Before 2015, serogroup W IMD caused < 5% (18/535 for 2010–2014) of all IMD; this increased to 40% (80/198) in 2017. Analyses of the whole genome sequences of the isolates showed that almost all serogroup W strains from 2015 to 2017 had the same finetype P1.5,2:F1–1 (122/134; 91%) and belonged to clonal complex 11 (cc11; 117/124; 94%). The incidence of serogroup W IMD not belonging to cc11 has remained stable over the last years with 1–5 cases per year, whereas the number of serogroup W cc11 cases started to increase in 2015 (Figure 1). 

**Figure 2:**
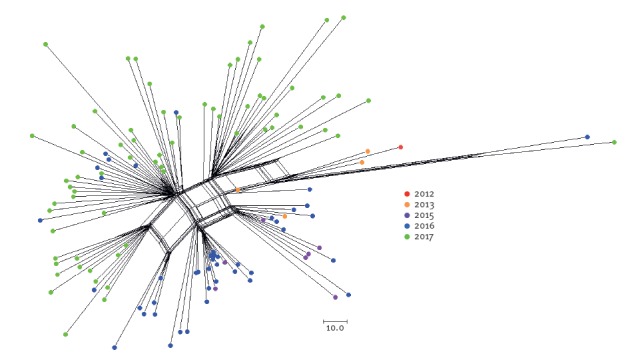
Neighbour-net phylogenetic network analysis of all available genomes of serogroup W clonal complex 11 isolates from the Netherlands, 2012–2017 (n = 121)

Figure 2 shows a cluster analysis of all available genome sequences of serogroup W cc11 meningococci isolated in 2012–2017 from Dutch patients. Isolates from the same year seem to cluster, i.e. the genetic distance between isolates is smaller within years than between years. Furthermore, within the same year several separate clusters can be discerned, which may suggest different introductions or more likely expansions of subclones. These genetic clusters were neither epidemiologically nor geographically associated.

Colours represent the years when the isolates were obtained. Genomes were compared using the PubMLST genome comparator tool using core genome multilocus sequence typing (cgMLST v1.0) [15]. The resulting distance matrices were visualised with SplitsTree4 version 4.13.1 [16].

**Figure 3:**
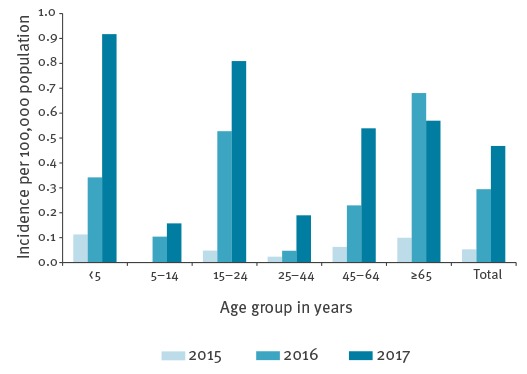
Incidence of serogroup W invasive meningococcal disease by age group in the Netherlands, 2015–2017 (n = 139)

In 2017, the incidence rate was highest in children < 5 years of age (0.92/100,000; n = 8), especially those < 2 year-old (2.0/100,000; n = 7 of whom four cases were < 1 year-old), and 15–24 year-olds (0.81/100,000; n = 17) (Figure 3). The incidence rate was also relatively high among older adults; the incidence started to increase from 45 to 50 years of age (0.4/100,000; n=5) up to an incidence of 1.6/100,000 in ≥ 80 year-olds in 2017 (n = 12). From 2015 to 2017, of 138 serogroup W IMD cases with known outcome, 17 (12%) died; six deceased cases were 15–24 year-olds. In comparison, eight of 215 (4%) serogroup B IMD cases died from 2015 to 2017. In 2017, of 73 serogroup W IMD patients with known clinical manifestation, 39 (53%) had septicaemia, 11 (15%) had meningitis, six (8%) had both septicaemia and meningitis, nine (12%) had pneumonia, and eight (11%) had other manifestations, including one case with necrotising fasciitis (case report in [1]). Meningitis, with or without septicaemia, was most prevalent in < 5 year-olds and 5–14 year-olds, while septicaemia was most prevalent in other age groups.

## Implementation of MenACWY vaccination

Based on advice from an expert meeting, the Dutch minister of Health decided in September 2017 to start implementing quadrivalent conjugate meningococcal vaccination (MenACWY) to control the increase in serogroup W IMD in the Netherlands. From May 2018 onwards, the MenC conjugate vaccine given at 14 months of age will be replaced by a MenACWY conjugate vaccine to give direct protection to this vulnerable age.

In addition, from October 2018 onwards, 13–14 year-olds will be offered a MenACWY vaccination. The aim is to give direct protection to this age group as the incidence and case fatality rate of serogroup W IMD is high in this group. Furthermore, as meningococcal carriage starts to increase from the age of 14 years [2], the expectation is that, by targeting this age group, carriage and transmission of serogroup W meningococci will be reduced and therefore disease in other age groups, e.g. children < 14 months of age and older adults, will be reduced as well. The epidemiological situation is continuously monitored to assess whether additional measures, e.g. extension to other age groups, need to be taken. All eligible children will receive a personal invitation for MenACWY vaccination – the invitation for 14-months-old children will be sent to their parents or guardians – from the National Institute for Public Health and the Environment. The adolescent vaccination will be administered by the Youth Health Care of the Municipal Health Service at dedicated group sessions. Adolescents who do not get their vaccination will receive a reminder within several weeks. Vaccination uptake will be closely monitored. Qualitative and quantitative research will be performed to evaluate acceptance of MenACWY vaccination in adolescents and their parents before and after introduction of vaccination.

## Discussion

Previous genomic analyses have shown that the serogroup W cc11 strain that is causing the current increase of IMD in the Netherlands, emerged in Brazil in 2003 and then spread to Argentina and Chile [3]. The strain emerged in the United Kingdom (UK) in 2009 causing an increase of serogroup W IMD in the UK and a further descendent of this strain, the so-called 2013 strain, expanded in the UK since 2013 [4,5]. The original and 2013 UK strains emerged in the Netherlands in 2012 and 2013, respectively, and the 2013 UK strain is currently causing the ongoing increase in serogroup W IMD [6]. Since 2015, also other European countries experience an increase in serogroup W IMD due to this specific cc11 strain including Denmark, France, Spain and Sweden [5,7–9], although the magnitude of the increase currently seems highest in the UK and the Netherlands. However, other countries may also see a further increase, and public health actions including vaccination could be considered.

Characteristics that seem typical for the current serogroup W IMD increase in Europe are the age distribution, with not only a high incidence in young children and adolescents but also in older adults, the high case fatality rate compared with other serogroups, and the clinical manifestation, with more septicaemia, less meningitis and more atypical manifestations including pneumonia and arthritis [4,6,8]. The UK reported a case series of teenagers with serogroup W IMD who presented with gastrointestinal symptoms and showed high case fatality rates [10]. A recent study of almost 12,000 IMD cases from 1991–2016 in France showed a higher case fatality rate in IMD patients with abdominal presentations compared to all IMD patients (24% vs. 10%). Serogroup W, and specifically cc11, was significantly more often the cause of IMD in patients with abdominal presentations [11].

Because of the ongoing increase of serogroup W IMD, the UK replaced the adolescent MenC conjugate vaccine for 13–14 year-olds and new university entrants by a MenACWY vaccine in the autumn of 2015 [12]. In addition, catch-up campaigns were implemented to offer the MenACWY vaccine to all 13–18 year-olds during 2015 to 2017. First results of the programme among school leavers showed a 69% decrease in observed cases (n = 6) compared with predicted cases by trend analysis (n = 19.4), despite only 36.6% coverage among persons who left school in 2015 [13]. Early estimated vaccine effectiveness using the screening method was 100% (95% confidence interval (CI): − 47 to 100%). Three other European countries, Austria, Greece and Italy, offer MenACWY vaccination to adolescents as part of their national vaccination programme, but not for reason of an increase in serogroup W IMD.

In contrast to the UK, only a single birth cohort of adolescents will be offered MenACWY vaccination in the Netherlands. Without a catch up campaign it will take several years before herd protection will be achieved. The goal of the current implementation of MenACWY vaccination is therefore not to control the increase of serogroup W IMD immediately but to prevent higher number of cases in the future.

A positive additional effect of introducing MenACWY vaccination is that adolescents will be boosted against serogroup C meningococci. From sero-epidemiological studies, it is estimated that the percentage of adolescents that currently has protective antibody levels against serogroup C meningococci is very low in the Netherlands [14]. The introduction of the MenACWY booster in adolescents will ensure high antibody levels in this age group, which is necessary to maintain herd protection against serogroup C IMD.

The Netherlands has a comprehensive surveillance system for IMD through linkage of statutory notifications received by the National Institute of Public Health and the Environment and laboratory data from the Netherlands Reference Laboratory for Bacterial Meningitis (NRLBM) (see [6] for more elaborate description). By means of this surveillance system comprising epidemiological, clinical and microbiological data, we continuously monitor the situation concerning the increase of serogroup W IMD to evaluate whether additional measures need to be taken.
